# EscaPRRS-ORF5: a structure-aware evolutionary framework for prioritizing immune escape-prone variants in porcine reproductive and respiratory syndrome virus

**DOI:** 10.1093/bioinformatics/btag529

**Published:** 2026-07-23

**Authors:** Ratul Chowdhury, Vaishnavey SR, Supantha Dey, Sakib Ferdous, Riza Danurdoro, Michael A Zeller

**Affiliations:** Department of Chemical and Biological Engineering, Iowa State University, Ames, IA 50011, United States; Department of Chemical and Biological Engineering, Iowa State University, Ames, IA 50011, United States; Department of Chemical and Biological Engineering, Iowa State University, Ames, IA 50011, United States; Department of Chemical and Biological Engineering, Iowa State University, Ames, IA 50011, United States; Department of Chemical and Biological Engineering, Iowa State University, Ames, IA 50011, United States; Department of Veterinary Diagnostic and Production Animal Medicine, College of Veterinary Medicine, Iowa State University, Ames, IA 50011, United States

## Abstract

**Motivation:**

Porcine Reproductive and Respiratory Syndrome Virus (PRRSV) is a rapidly evolving RNA virus causing significant economic losses, posing a formidable challenge to vaccine efficacy due to its high mutational variability and immune escape. As the viral mutants evolve, their ability to sustain in population is driven by a range of host biology factors such as receptor binding, fusion, and uncoating. Existing tools that predict viral fitness and escape propensities rely heavily on extensive, up-to-date sequence data and lack integration of biochemical host interactions, limiting mechanistic understanding of the mutational landscape. We introduce Esca, a sequence-only toolchain framework that identifies immune escape-prone residues by exhaustively scanning each residue position for all amino acid substitutions using a Bayesian Variational Autoencoder (VAE) trained on protein language model embeddings. We demonstrate Esca on the GP5(ORF5) glycoprotein of PRRSV (EscaPRRS-ORF5) by training on ESM-2 embeddings of 32 146 GP5 sequences (2015–2022) spanning 140 sub-lineages.

**Results:**

Despite being trained only on GP5 sequence data, EscaPRRS-ORF5 recovered 85.7% of the surface-exposed receptor binding interfaces as escape-prone regions. We use a mutation-sensitive fitness scoring scheme that goes beyond Hamming distances, to predict antibody escape tendencies, supporting surveillance of (re) emerging PRRSV variants. We do not claim that ORF5 alone captures PRRSV evolution or serves as a surveillance endpoint; rather, Esca offers a scalable path toward whole-genome, structure-aware surveillance.

**Availability and implementation:**

EscaPRRS-ORF5 is freely available at https://doi.org/10.6084/m9.figshare.32661033 with an interactive Colab notebook at https://colab.research.google.com/drive/1TEgzAhPwvNAZ01VXeJbIFibfri2jnDA5? usp=sharing.

## 1 Introduction

With over $1.2 billion annual losses in the United States, Porcine Reproductive and Respiratory Virus (PRRSV) poses a significant economic challenge for the swine industry, with implications to food security and human health. Vaccine design efforts for PRRSV have consistently been challenged by the virus’s complex and tightly controlled infection mechanisms. Traditional approaches that identify antiviral targets via directed evolution are often insufficient to keep pace with its extensive plasticity (Baker *et al.* 2025). Thus, semi-rational designs aware of host-antigen interactions and dynamic, disordered regions of viral proteins are suggested. The emergence of protein language model-based tools (Cheng *et al.* 2023, Ito *et al.* 2025), which implicitly capture molecular interaction landscapes, enables predictions of epidemiological fitness propensities of viral variants.

In metazoan cells, antibodies neutralize pathogens by blocking viral entry, propagation, and immune evasion, often by binding to Receptor Binding Domains (RBDs) and interfering with host-virus interactions (Chan *et al.* 2025). However, viruses evade these defenses through mutations in surface glycoproteins that alter charge distribution and structural complementarity. Consequently, immune escape undermines vaccine efficacy, as evolving viral surfaces diminish antibody binding to designed immunogens (Li *et al.* 2025).

Contemporary PRRSV is classified into *Betaarterivirus europense* (PRRSV-1) and *Betaarterivirus americanese* (PRRSV-2), colloquially referred to as the European PRRSV and American PRRSV respectively (ICTV Report), reflecting substantial genetic divergence. Although PRRSV-1 is present in North America, it accounts for ≤1% of annual detections since 2015 based on Swine Disease Reporting System data (Trevisan *et al.* 2020), and remains associated with lower clinical impact. In contrast, PRRSV-2 comprises > 99% of PRRSV in the U.S and is responsible for significant disease burden and mortality, including the emergence “highly pathogenic” (HP) strains affecting southeast Asia and China (Tong *et al.* 2007). The standard control strategy for PRRSV is to use modified live vaccines that contain an attenuated virus. In the United States there are currently 6 licensed PRRSV-2 vaccine products available (Rawal *et al.* 2022, Li *et al*. 2024). While PRRSV-1 is a potential future concern, there are no currently approved vaccines available, due to the low detection rate and lesser clinical signs presented by this species. Accordingly, this study focusses on ORF5-linked fitness scoring of PRRSV-2, given its endemic persistence and high pathogenicity.

PRRSV enters host cells through a multistep process mediated by interactions between viral glycoproteins and receptors on porcine alveolar macrophages (PAMs) (Jiang *et al.* 2025). Its envelope contains two main glycoprotein complexes: the GP5-M heterodimer, responsible for receptor binding, and the GP2-GP3-GP4 trimer, which stabilizes the virion and enhances infectivity (Gao and Wen 2025). Viral entry occurs through receptor mediated endocytosis, involving binding, clustering of receptor-antigen complexes and membrane reorganization (Wei *et al.* 2020). Cryo-electron microscopy shows PRRSV virions as 55 nm spherical particles with small ectodomains (2 nm) of GP5-M proteins.

Of the ten open reading frames (ORFs), ORF1a encodes the polyprotein 1a, which is processed into 14 non-structural proteins and four proteinases, while ORF2-ORF7 encode structural proteins including GP2-GP5, membrane proteins and the nucleocapsid (Meng 2000). ORF5 is the most studied region of PRRSV and is seen to be the most variable region that harbors antibody neutralizing epitopes (Jian *et al.* 2025, VanderWaal *et al.* 2025, Cotaquispe Nalvarte *et al.* 2026). This 200-residue GP5 glycoprotein is known to affect neutralization of antibody targets, impairing cross-protection by immune responses raised against different strains (Wei *et al.* 2012). While GP5 solely does not govern viral tropism, available literature (Gao and Wen 2025) on porcine receptor interactions to PRRSV-2 indicated the GP5 mediates weak attachment to Heparansulfate that possibly positions the virus for subsequent high affinity binding with Sialoadhesin where the proteoglycans attach to the M-GP5 complex. Additionally, CD163 receptor is widely reported to be involved in uncoating viral particles to activate the infector pathway. Nontarget cells expressing both Sialoadhesin and CD163 are found more susceptible than those that express only CD163 (Van Gorp *et al.* 2008). In-vitro studies demonstrate that the expression of CD163 alone is sufficient for infection and replication as CD163 is solely responsible for disassembly of the viral envelope and capsid, causing conformational changes in GP2-GP4 that destabilizes the membrane-nucleocapsid interface (Xu *et al.* 2022, Zhu *et al.* 2023). Complete knockout of CD163 in porcine gestational pigs proved resistant to PRRSV infection with no fetal pathology while WT fetuses were up to 92% PRRSV positive. This is because CD163 blocks the infection pathway in maternal macrophages that normally carry the virus across the placenta (Prather *et al.* 2017). Several studies report improved resistance across all PRRSV genotypes in CD163 knockout pigs (Yang *et al.* 2018, Guo *et al.* 2019). Hence, a high affinity binding of one or more envelope proteins of PRRSV to porcine receptors is indicated to govern the overall tropism of PRRSV-2.

Our previous work (Dey *et al.* 2024) on PRRSV epitope atlas serves as a roadmap for structural characterization of host-pathogen interactions beyond the CD163 receptor. Given the immunodominance and abundant availability of GP5 sequences over other viral glycoproteins, we analyze all possible single point mutants that potentially obscure neutralizing epitopes, alter local charge distribution and sterically block antibodies to confer viral escape. To this end, we have developed a structurally guided escape-variant predictor CTRL-V and demonstrated its efficacy in predicting mutations observed in SARS-CoV-2 variants of concern starting with the parental sequence for which the receptor binding domain was co-crystallized with the human ACE2 protein and commercial Ly-CoV1404 antibodies (Teoh *et al.* 2025).

Anticipating escape tendencies using Machine Learning (ML) based computational tools is viewed as a promising approach to identify and predict pathogenic variants (Chen *et al.* 2021, Teoh *et al.* 2025). The EVE model (Frazer *et al.* 2021) uses a Bayesian Variational Autoencoder (VAE) on the homologous protein sequences, represented as one-hot encodings to capture evolutionary constraints that maintain protein fitness. Thus, variants with a lower modeled probability are predicted as less fit (deleterious). EVEscape (Thadani *et al.* 2023) extends these fitness predictions by integrating them with structure and biophysical constraints to score escape propensities in SARS-CoV-2 spike protein. The integration of protein language models into genomic surveillance pipelines has proven useful for real-time monitoring and characterization of emerging variants(Lytras *et al.* 2025). This work (Esca) fundamentally expands the scope and power of EVEscape scoring capabilities to predict critical future variants by replacing the one hot amino acid encoding with state-of-the-art ESM-2 (https://huggingface.co/facebook/esm2_t33_650M_UR50D) residue embeddings thus providing a structural basis of interpreting these variants through receptor interactions. ESM residue embeddings carry pre-trained evolutionary patterns from millions of protein sequences and even capture spatial relationships between amino acid pairs. This allows us to accurately assess mutational effects to pinpoint functionally important loci for vaccine targets without the need for explicit sequence alignments and experimentally resolved structures.

Here, we demonstrate Esca (Escape propensity prediction) for PRRSV-2 variants (EscaPRRS), a structure-aware fitness and escape prediction model, trained on ESM-2 embeddings using a Bayesian VAE. We demonstrate EscaPRRS’s fidelity for a robust understanding of GP5 glycoprotein, by training on 32 146 PRRSV-2 GP5 protein sequences (encoded by ORF5 genes) from the United States from 2015 to 2022, referred to as EscaPRRS-ORF5 hereon. Although trained on local sequences, ESM embeddings capture structural and evolutionary context, generalizing the validity of EscaPRRS-ORF5 scores for all (5291) publicly available PRRSV-2 GP5 sequences. We demonstrate the biological relevance of EscaPRRS-ORF5 antibody escape scores for PRRSV GP5 mutants, providing insights to competitive relative binding affinities at interfacial hotspots for seven different porcine receptor proteins. These receptors include: CD151, CD209, CD163, Sialoadhesin, Heparansulfate sulfotransferase, Vimentin, and MYH-9, identified through our epitope atlas mapping endeavor (Dey *et al.* 2024). EscaPRRS-ORF5 residue level escape score predictions accurately capture evolutionary patterns and pinpointed 85.7% of the surface exposed receptor-binding interfaces as escape-prone regions, while preserving seasonal and lineage-specific patterns arising from population sampling. In recognizing the limitations inherent in conventional fitness models that rely solely on sequence similarity patterns, we underscore the value of integrating protein language model embeddings to capture both evolutionary and structural signals that affect the fitness landscape of complete sequences of emerging variants.

## 2 Methodology: Esca scoring framework

**Figure 1:**
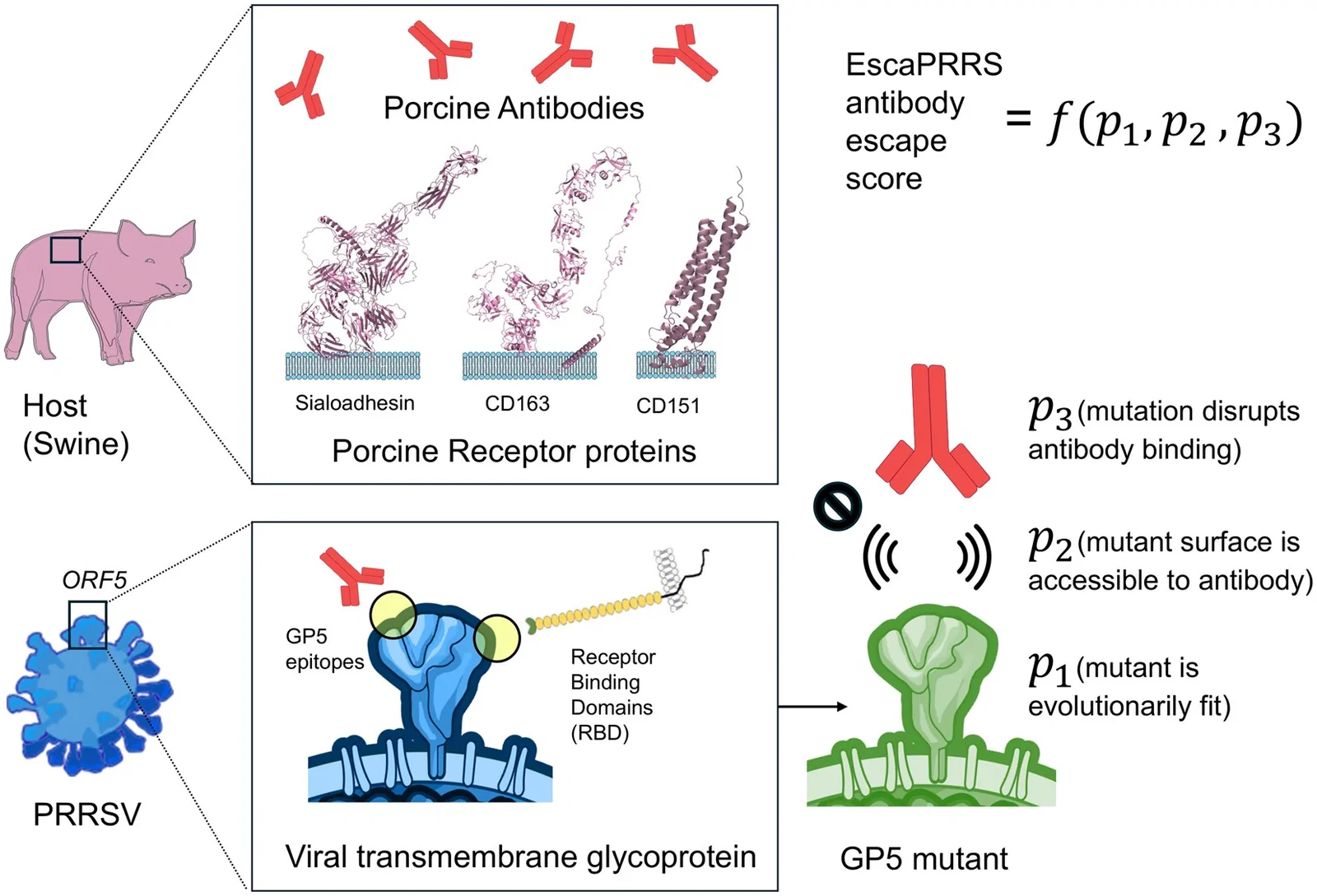
Antibody escape scoring for a single point PRRSV mutant with EscaPRRS-ORF5. The current implementation employs a multiplicative combination of the individual component scores. The genetic construct of GP5 is indicated as ORF5. Illustrations adapted from NIAID NIH BioArt Source #465 and #652.

Esca combines evolutionary information with local structural and physiochemical changes to score antibody escape propensity across the complete mutational landscape of any viral protein. Figure 1 illustrates the structure-function basis of EscaPRRS antibody escape propensity scoring at the host-receptor-antibody interface, highlighting its ability to complement experimental efforts that typically assay only a limited subset of PRRSV GP5 variants against available monoclonal antibodies.

Sequence distributions under natural selection have been modeled (Pugh *et al.* 2025) to obey a Boltzmann distribution profile, where the log-likelihood of a sequence is proportional to its evolutionary fitness, providing an unbiased zero-shot proxy for mutational tolerance. In EscaPRRS, fitness is estimated using a Bayesian VAE framework adapted from EVE. Instead of one-hot encodings, input sequences are represented using ESM-2 embeddings, which capture contextual and evolutionary features. These embeddings are processed through a probabilistic latent space, where the encoder is trained to minimize the evidence lower bound (ELBO) with a Kullback–Leibler (KL) divergence penalty to enforce consistency with the prior distribution.

To further enhance biological realism, EscaPRRS incorporates a 1D convolutional layer in its output, capturing local dependencies across sequence positions. This improves upon the positionally independent one-hot encoding scheme used in EVE (Fig. S1, available as supplementary data at *Bioinformatics* online), enabling better generalization to unseen sequences with conserved structural motifs. The sensitivity of ESM-2 embeddings to single-point mutations supports downstream analyses, such as ELBO-based evolutionary indices and identification of functionally important residues at virus–host interfaces.

Antibody escape is governed by three complementary requirements: (i) the variant must remain evolutionarily viable, (ii) the mutation site must be structurally accessible to antibodies, and (iii) the mutation must induce sufficient physicochemical change to disrupt binding. Accordingly, EscaPRRS models escape propensity using three corresponding components: fitness, surface accessibility, and chemical dissimilarity.

Surface accessibility is quantified using the weighted contact number (WCN) (Lin *et al.* 2008), defined as the sum of inverse squared distances between all residue pairs in a protein structure. Distances are computed between centers of mass of residue side chains, ensuring that nearby residues contribute more strongly. Lower local packing density corresponds to higher surface exposure, making WCN an effective proxy for identifying residues accessible to antibody binding.

Chemical dissimilarity is computed using a charge–hydrophobicity metric, following the EVEscape (Thadani *et al.* 2023) paradigm, based on differences in Eisenberg–Weiss hydrophobicity and residue charge. This captures the extent to which a mutation perturbs the local physicochemical environment at potential antibody binding sites.

These three components are transformed into probability distributions using Boltzmann-like scaling (SoftMax normalization) with temperature parameters controlling sensitivity. The resulting probabilities—fitness (P_1_) accessibility (P_2_), and dissimilarity (P_3_) are combined multiplicatively to yield the overall escape score.

This multiplicative formulation represents a joint likelihood in which the three components act as complementary and conditionally independent requirements for immune escape. It enforces an AND-like interaction, ensuring that mutations must be simultaneously viable, exposed, and antigenically novel to achieve high escape propensity. Consequently, a low probability in any single component proportionally suppresses the overall score, resulting in robust, biologically consistent, and interpretable predictions across complex mutational landscapes.

**Figure 2:**
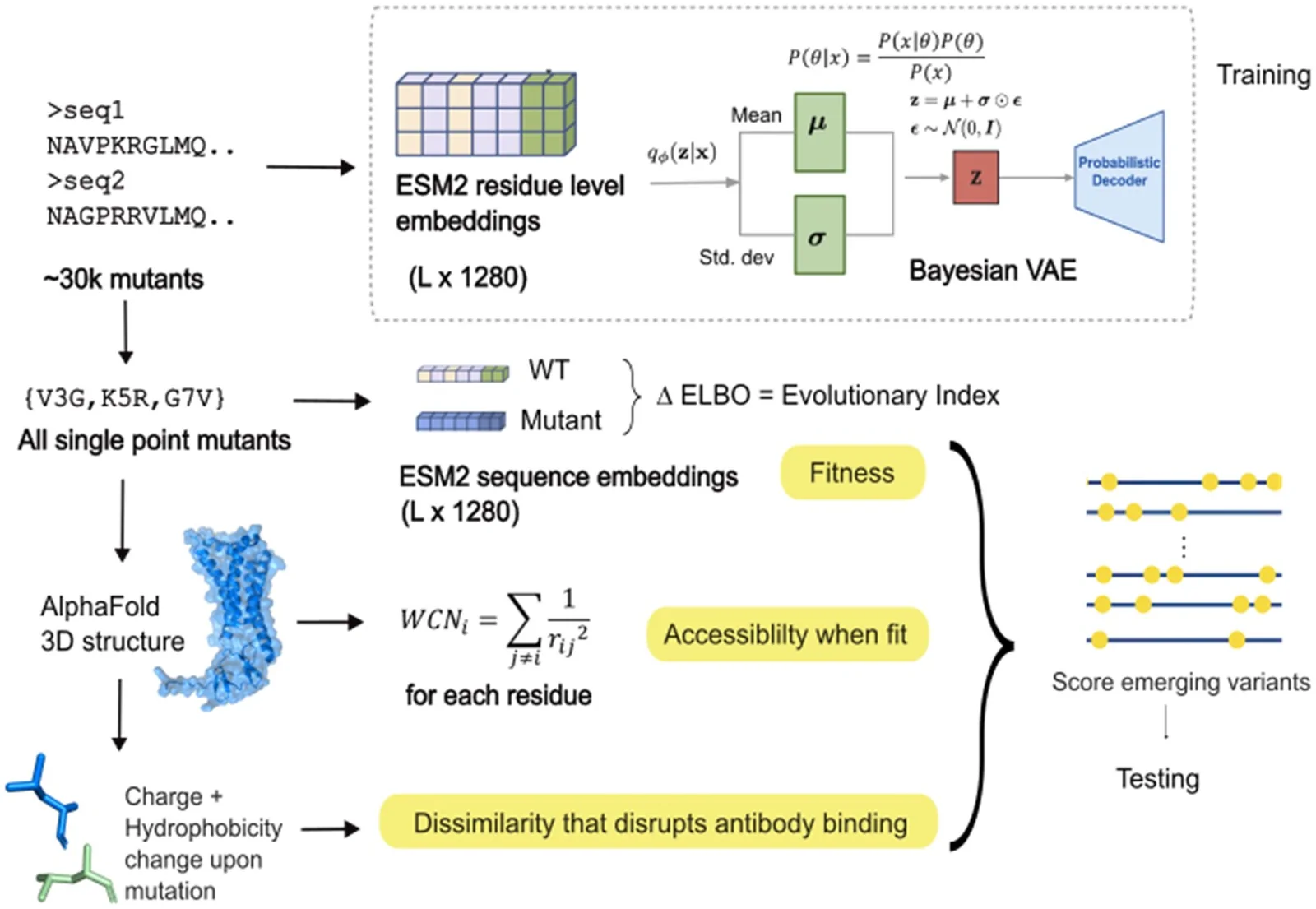
The EscaPRRS-ORF5 (generally Esca) escape propensity scoring of emerging sequences incorporating fitness, surface accessibility and charge dissimilarity. ESM-2 embeddings from PRRSV GP5 sequences are trained on a Bayesian Autoencoder that captures variational inference from sequence distributions as in EVE30, to estimate evolutionary fitness of all single point mutants from the wild type. These fitness calculations are enriched with surface avidity and dissimilarity metrics to predict relative escape propensities. The single point mutation effects are aggregated to score emerging variants and sequence constructs for escape propensities.

As shown in Fig. 2, the overall escape score is calculated as a product of fitness, accessibility and dissimilarity scores with suitable logistic temperature scaling factors $(T_{f}\equiv T_{1}{,\;T}_{a}\equiv T_{2}$, and $T_{d}\equiv T_{3}),\;$referred to as escape factors as shown in Equation 1.


(1) $$\mathrm{EscaPRRS}–\mathrm{ORF}5\;\mathrm{Score}=\mathrm{logistic}(\frac{\mathrm{Fitness}}{T_{f}})\times \mathrm{logistic}(\frac{\mathrm{Accessiblity}}{T_{a}})\times \;\mathrm{logistic}(\frac{\mathrm{Dissimilarity}}{T_{d}})=\;{\;P}_{1}\times P_{2}\times P_{3}$$

(2) $$P_{i}=\frac{\;\text{exp}(-\;\frac{{\mathrm{score}}_{j}}{T_{j}})}{\sum_{j}\;\text{exp}(-\;\frac{{\mathrm{score}}_{j}}{T_{j}})},$$

Where i  ∈ {1=fitness, 2=accessibility, 3=dissimilarity} ⊂ escape components and $T_{i}$ denotes the temperature (scaling) parameter associated with each escape component *i*, (1 = fitness, 2 = accessibility, and 3 = dissimilarity) which modulates the sensitivity of the calculated probability to the corresponding *score_i_* that represents the raw score calculated for that component. The denominator ensures the probabilities are normalized to sum to unity. Details on training dataset curation and autoencoder training are provided in the Supplementary Methods S1, available as supplementary data at *Bioinformatics* online.

## 3 Results and discussion

### 3.1. Complete fitness landscape for PRRSV GP5 mutants using EscaPRRS-ORF5

A major component of an escape prediction framework is to demarcate relative fitness of circulating variants as soon as they emerge, to aid parallel efforts on escape-proof vaccine design, as demonstrated in our CTRL-V (Teoh *et al.* 2025) effort. In other RNA viruses like in HIV and SARS-Cov-2, the replicative fitness of a variant in the sequence population can be compromised in high antibody escape variants but its fitness is reestablished by means of structural and functional changes at the secondary residues not directly linked to primary escape mutation sites (Lynch *et al.* 2015, Teruel *et al.* 2025). Thus, capturing the fitness landscape beyond phylogenetic context is important to identify critical structural domains conferring immune escape and those enabling fitness.

**Figure 3:**
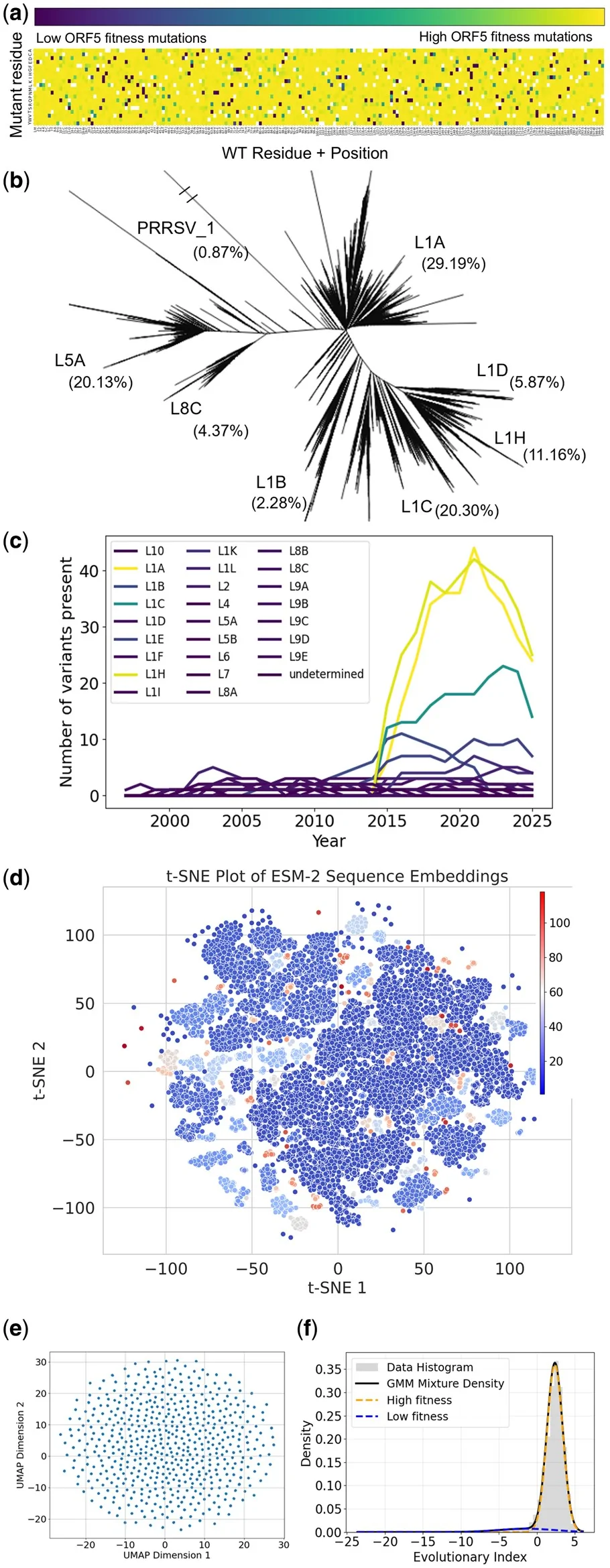
(a) EscaPRRS-ORF5 fitness calculation for all possible single point mutants to a consensus PRSSV2-GP5 reference sequence captures specific residue position and choices that compromise the evolutionary fitness of the resulting single point mutant. This is calculated from the latent space trained on 32 146 sequences. (b) IQ-TREE Maximum likelihood phylogenetic tree of the sequences used in this study annotated with nine lineages of PRRSV-2 and the percentage of sequences within each lineage. (c) Population occurrence trends in PRRSV-2 lineages publicly reported by VanderWaal *et al*. (d) t-SNE visualization of ESM-2 embeddings derived from 32 146 protein sequences in the training dataset color coded based on DBSCAN (Density-based spatial clustering of applications with noise) cluster numbers (e) UMAP (Uniform Manifold Approximation and Projection) visualization of the latent space learned by the VAE on EscaPRRS-ORF5 capturing smoothened sequence variability. (f) 2-component Gaussian Mixture Model (GMM) fit to the evolutionary index distribution to allow probabilistic separation between ORF5 fitness classes.

ESM-2 captures evolutionary patterns by storing coevolutionary statistics of interacting sequence parameters and pairwise residue dependencies, implicitly learning local motifs and their separation distances (Zhang *et al.* 2024). The Bayesian Variational Autoencoder trained on ESM-2 embeddings for 32 146 PRRSV-2 sequences under a fixed hyperparameter configuration (described in Table S1, available as supplementary data at *Bioinformatics* online) was used to calculate the departure of the resultant sequence distribution for all single point mutants from the reference sequence. This is used to calculate the fitness as the log likelihood of every mutant, visualized in Fig. 3, panel(a). The fitness values are then parsed into a two-component Gaussian Mixture Model (GMM) to classify into low and high ORF5-linked fitness. The posterior fitness probabilities are computed with quantified uncertainty values for downstream analyses.

It is important for a mutation to be fit over its evolutionary counterparts to survive in time and for its surface to be available for binding to the host receptor, and the antibodies. From our dataset, we observed 9 sequences consistently re-emerging 100–870 times from 2015 to 2022, implying superior replicative fitness, transmission potential and immune evasion under shared selective pressures. This is supported by low gamma shape parameter of the maximum likelihood phylogenetic tree (α=0.65) (Fig. 3, panel (b), Supplementary Methods S2, available as supplementary data at *Bioinformatics* online), showing a prevalence of high ORF5-fitness variants that have nearly zero evolution rates, with a few hotspots driving rapid evolution under selection. We can also see a higher number of high ORF5-fitness variants over time publicly reported by VanderWaal *et al.* (2025) (Fig. 3, panel (c)).

Thus, capturing the fitness landscape beyond phylogenetic context is important to identify critical structural domains conferring immune escape and those enabling fitness. ESM-2 captures evolutionary patterns by storing coevolutionary statistics of interacting sequence parameters and pairwise residue dependencies, implicitly learning local motifs and their separation distances (Zhang *et al.* 2024). The Bayesian Variational Autoencoder trained on ESM-2 embeddings for 32 146 PRRSV-2 sequences under a fixed hyperparameter configuration (described in Supplementary Table S1, available as supplementary data at *Bioinformatics* online) was used to calculate the departure of the resultant sequence distribution for all single point mutants from the reference sequence. This is used to calculate the fitness as the log likelihood of every mutant, visualized in Fig. 3, panel(a). The fitness values are then parsed into a two-component Gaussian Mixture Model (GMM) to classify into low and high ORF5-linked fitness. The posterior fitness probabilities are computed with quantified uncertainty values for downstream analyses.

It is important for a mutation to be fit over its evolutionary counterparts to survive in time and for its surface to be available for binding to the host receptor, and the antibodies. From our dataset, we observed 9 sequences consistently re-emerge 100–870 times from 2015 to 2021, implying superior replicative fitness, transmission potential and immune evasion under shared selective pressures. This is supported by low gamma shape parameter of the maximum likelihood phylogenetic tree (α=0.65) (Fig. 3, panel (b), Supplementary Methods S2, available as supplementary data at *Bioinformatics* online), showing a prevalence of high ORF5-fitness variants that have nearly zero evolution rates, with a few hotspots driving rapid evolution under selection. We can also see a higher number of high ORF5-fitness variants over time publicly reported by VanderWaal *et al.* (2025) (Fig. 3, panel (c)).

### 3.2. EscaPRRS-ORF5 for antigenic escape landscape

While phylogeny can influence apparent virulence of co-evolving strains, evolutionary classification is only a partial proxy for virulence and genetically homologous variants do not directly translate to immunological cross protection (Geoghegan and Holmes 2018). To this end, antibody escape propensity predictions offer a powerful refinement of the antigenic dimension to identify variants of concern (VOCs), forecast mutations with high escape potential from monoclonal and polyclonal sera, and monitor the combinatorial fitness landscape (Starr *et al.* 2021).

**Figure 4:**
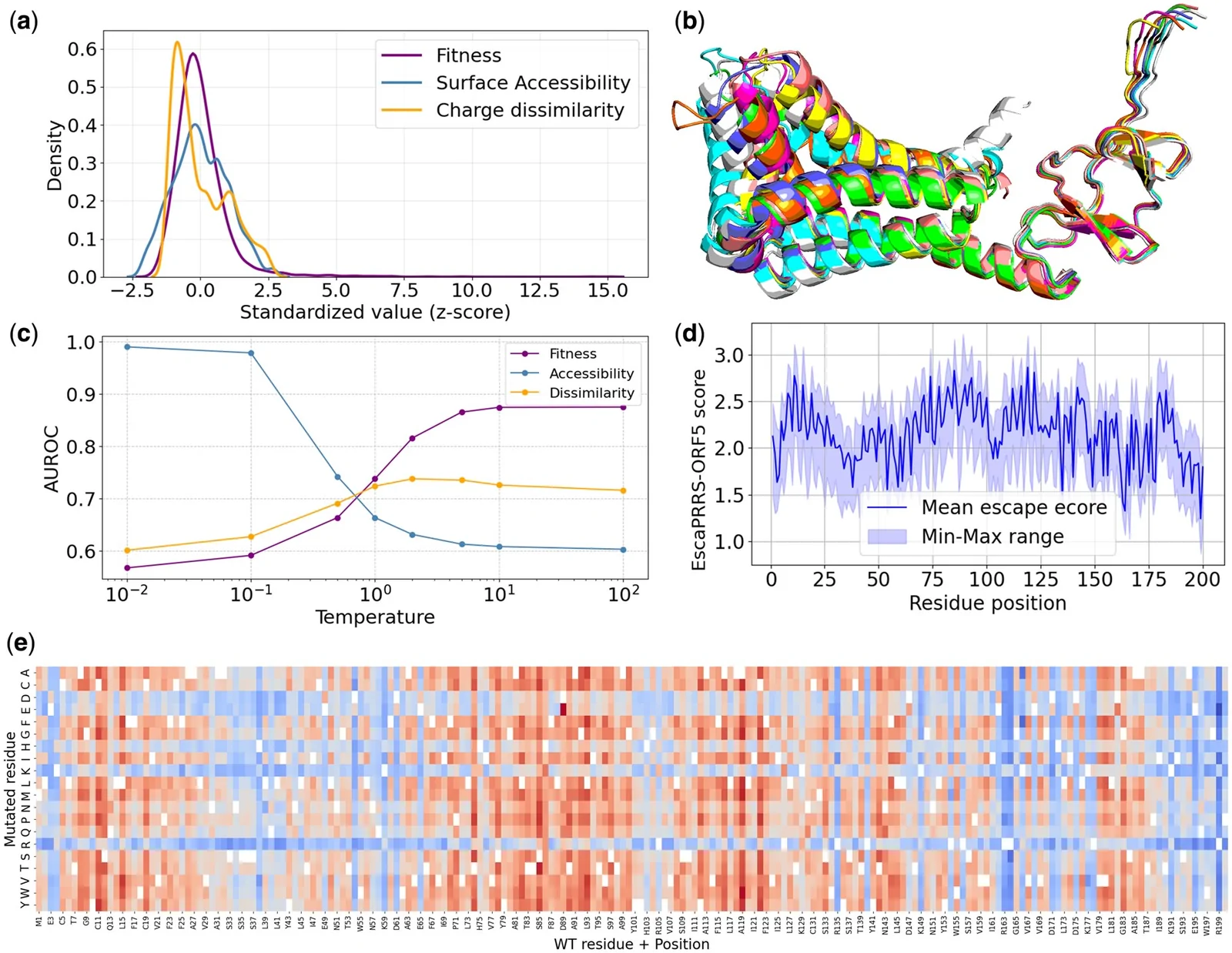
(a) Comparison of standardized distribution of ORF5-fitness, surface accessibility score, combined charge-hydrophobicity metric for the complete single point mutational landscape for PRRSV-GP5. (b) Overlay of AlphaFold3 predicted high confidence structures (pTM > 0.5) for GP5 glycoprotein of PRRSV-2 across different lineages (L1A-L1F, L5A, L8C) observed in the training dataset, indicating a structurally conserved backbone (Relative RMSD < 1.5 Å) used to calculate weighted contact number for each residue position. (c) Ablation analysis of EscaPRRS-ORF5 showing how per-factor temperature scaling affects the self-consistency of the combined EscaPRRS score. (d) Line plot of mean and variable EscaPRRS scores along the sequence length of PRRSV-GP5. (e) Heatmap of calculated EscaPRRS-ORF5 antibody escape propensities for all possible single point mutations from the reference PRRSV-GP5 sequence. Red regions indicate high escape propensity while blue regions indicate lower escape propensity.

For evolutionary fitter mutants, immune escape is conferred by the change in the local structure and charge distribution that render the interfacial surface incompatible/unstable for antibody binding. As shown in Equation 2, the EscaPRRS protocol integrates these features to calculate the antibody escape propensity score using a combined charged-hydrophobicity metric and the surface accessibility. Figure 4, panel (a) shows the standardized distribution of each of the three escape conferring factors for the complete mutational landscape of PRRSV-GP5.

We use the AlphaFold3 (Abramson *et al.* 2024) generated backbone structure of the consensus PRRSV-GP5 sequence to calculate the weighted contact number for each residue as a scalable surface accessibility metric, owing to the highly conserved backbone structure across different PRRSV lineage sequences, as point mutations do not drastically influence the global fold of the glycoprotein, seen in Fig. 4, panel (b).

The escape conferring factors including evolutionary fitness, surface accessibility and charge dissimilarity are subjected to ablation analysis, which helps adjust the inherent trade-off between evolutionary plausibility and immune evasion, which is uniquely distributed for each viral protein based on its structural rigidity and evolutionary plasticity. Youssef *et al.* (2024) demonstrated hyperparameter searches to optimize escape factors by varying the temperature across a range of values and reported the resulting AUROC values for different viral protein families including flu, HIV, and SARS-Cov-2. Higher AUROC values corresponded to the efficacy of the escape factor setting in the model to predict true escape mutations while avoiding false positives.

In our setting, because no experimental escape labels were available for PRRSV GP5, ablation was performed in an unsupervised manner, and the reported AUROC values quantify self-consistency with a baseline EscaPRRS ranking, rather than accuracy against ground truth. Our ablation results, as seen in Fig. 4, panel (c) revealed that structural accessibility reflecting solvent exposure of residues, is the most critical factor in EscaPRRS-ORF5 performance. Adjusting the temperature scaling for accessibility resulted in a 38.71% performance decrease, underscoring its strong sensitivity. In contrast, the fitness feature (mutational tolerance) showed a 30.75% increased performance, and dissimilarity (biochemical divergence from wild type) had the smallest effect with 13.66% improvement. Furthermore, the *R*^2^ score decreased most significantly to 0.1717 upon removal of the accessibility feature, confirming its essential role in PRRSV GP5 mutational escape prediction. The minimal impact observed when removing fitness (*R*^2^ = 0.9006) and dissimilarity (*R*^2^ = 0.9042) suggests that structural features predominantly govern escape tendencies, indicating that immune escape mutations are primarily located at structurally accessible sites, likely antibody epitopes, rather than being driven by selection for enhanced receptor binding or altered host mimicry. This is analogous to hypermutability of CDR3 surface exposed loops of antibodies which are constantly under selection pressure to bind to a rapidly evolving antigen (Chowdhury *et al.* 2018, Boorla *et al.* 2023).

Inspecting unsupervised temperature-response ablation curves, the fitness plateaus beyond a scaling of 10, while accessibility feature drops sharply after 0.5 and dissimilarity peaks around 2–5. Thus, pushing temperature scaling factors beyond these regions yields diminishing returns and can induce numerical saturation. Since the default escape factors (in EVEscape) were initially set to 1, 1, and 2, we performed a targeted adjustment of temperatures in EscaPRRS-ORF5 based on this ablation analysis with an unsupervised objective that favors stability and entropy for a balanced scoring of mutational escape in EscaPRRS-ORF5. Figure 4, panel (d) shows the average EscaPRRS-ORF5 scores for each residue position in the PRRSV-GP5 sequence, with panel (e) showing the complete mutational landscape for all single point mutants colored based on EscaPRRS-ORF5 scores calculated at the adjusted escape scaling factors (10,2,1.5 for fitness, accessibility, and dissimilarity respectively). Some of the low escape (blue-hued) regions are reported in the literature as GP5 residues prone to antibody neutralization, including N51, N29, N32, N44 (Vu *et al.* 2011). Positions 37 through 44 are reported to induce neutralizing antibody in addition to V102 (Han *et al.* 2019).

### 3.3. Immune escape propensities at top receptor binding hotspots

The normalized mutation-level EscaPRRS-ORF5 scores grouped by residue positions were used to calculate site average antibody binding escape propensities. Our previous work (Dey *et al.* 2024) annotated binding interface residues of all glycoproteins, membrane envelope proteins and non-structural proteins to seven different porcine receptors consistent across different docking platforms including GRAMMDock (Singh *et al.* 2024), HADDOCK3 (Giulini *et al.* 2025) and ClusPro (Kozakov *et al.* 2017) to characterize the epitope atlas of PRRSV-2.

**Figure 5:**
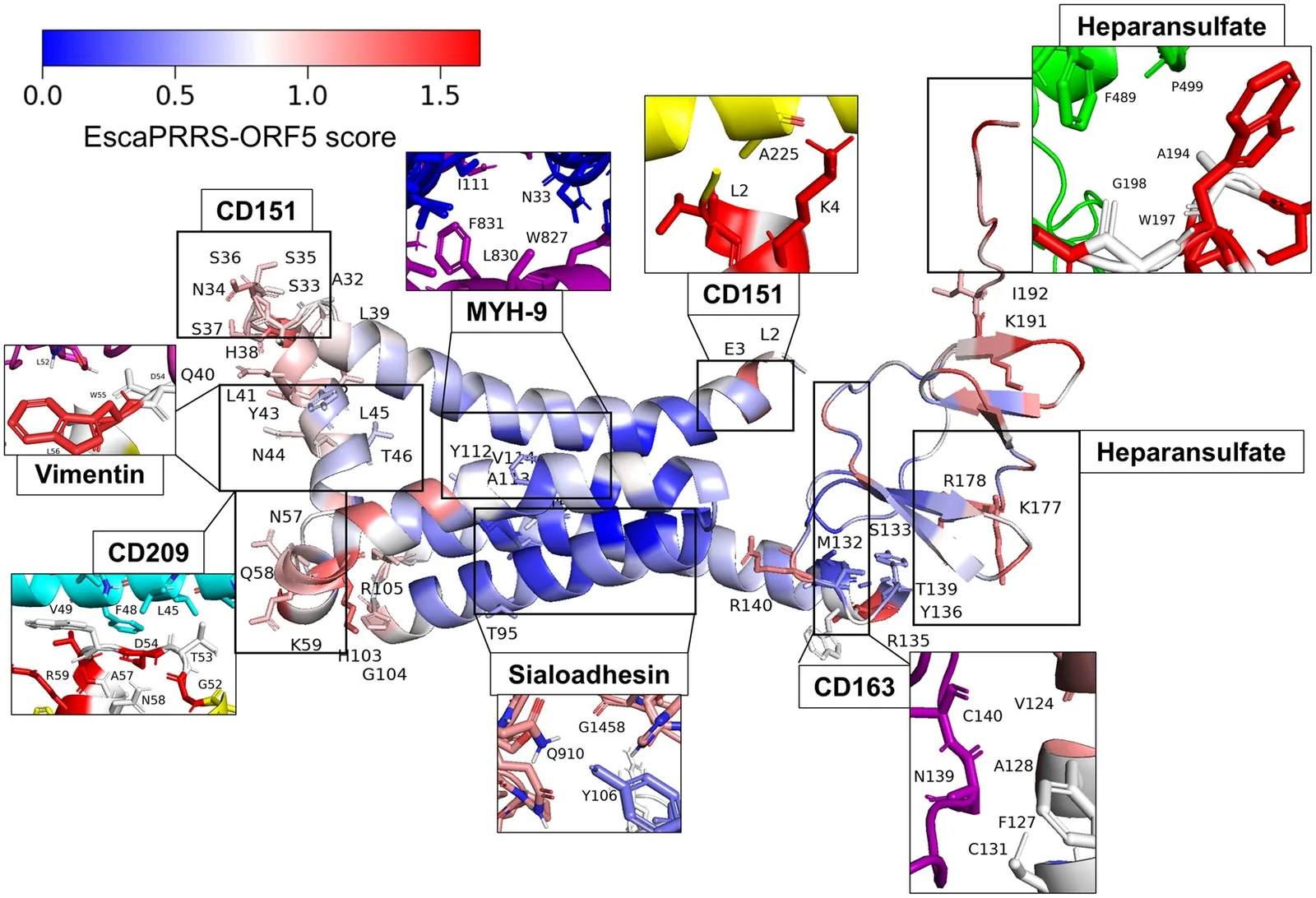
3D structure of PRRSV GP5 annotated with residues known to interact with seven porcine receptor proteins (yellow: CD151, cyan: CD209, purple: CD163, magenta: vimentin, peach: Sialoadhesin, green: Heparansulfate sulfotransferase, violet: MYH-9). GP5 Residue colors indicate EscaPRRS-ORF5 scores where red indicates higher escape propensity and blue corresponds to lower escape propensity.

These docked complexes of seven receptors with GP5, namely CD151, CD209, CD163, Sialoadhesin, Heparansulfate sulfotransferase, and MYH-9 indicated high EscaPRRS-ORF5 escape propensity regions interface with critical binding hotspots with porcine receptors, as seen in Fig. 5.

**Figure 6:**
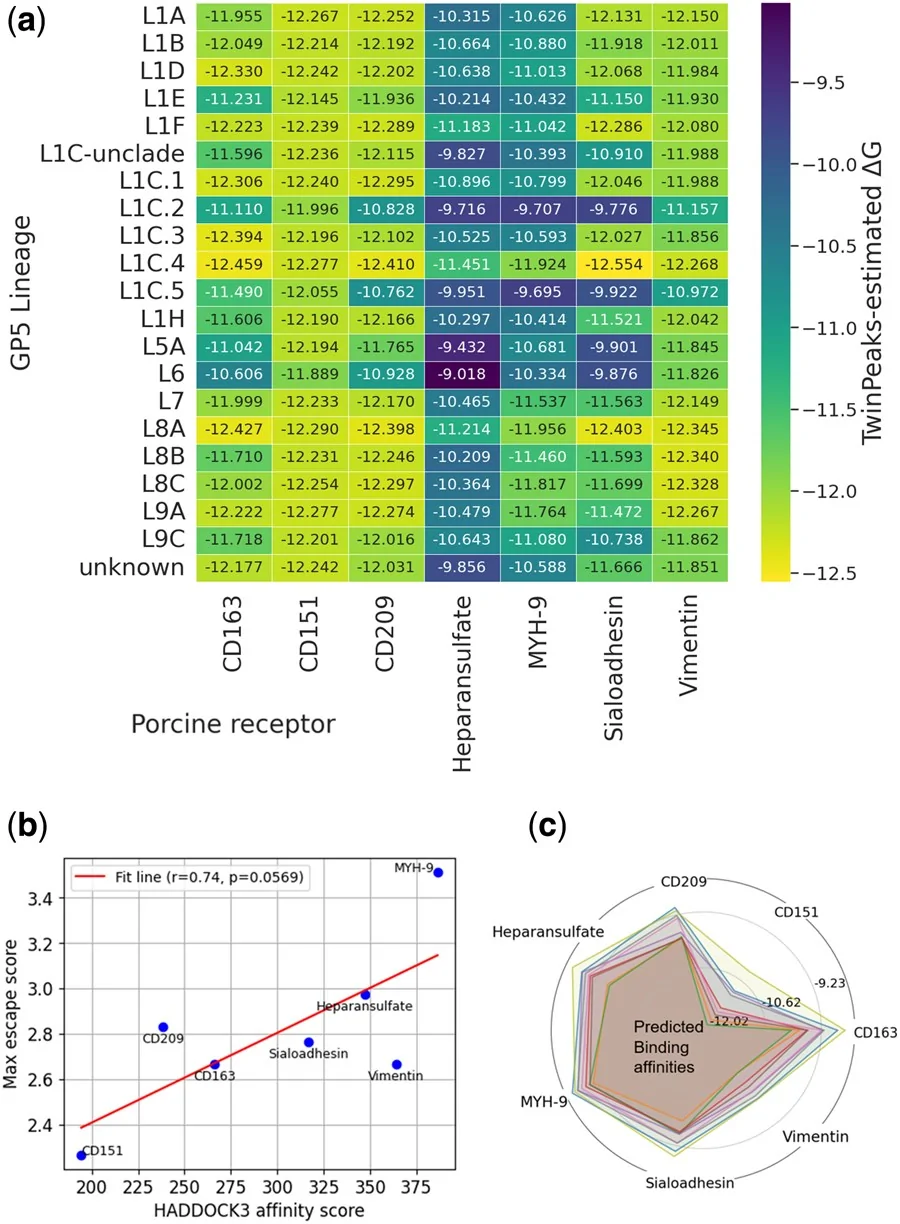
(a) Structure free binding affinity calculation using TwinPeaks (Dey *et al*. 2024) for GP5 complexes with seven porcine receptors across 13 different lineages of PRRSV-2 classified based on ORF5. **(**b) Correlation between maximum EscaPRRS-ORF5 antibody escape scores at the interfacial residues with HADDDOCK3 binding affinities for the reference GP5 sequence-receptor complexes. Residue-wise scores at the interfacial residues are provided in Table S2, available as supplementary data at *Bioinformatics* online. (c) Sequence-only binding affinity prediction for six unique sequences belonging to different ESM-2 embedding space t-SNE clusters on seven porcine receptors reveal similar trends with GP5 showing a strong binding affinity for CD151 and weaker binding to Heparansulfate sulfotransferase, as described in the introduction. Binding affinity scores for individual GP5-sequence receptor pairs are given in Table S3, available as supplementary data at *Bioinformatics* online.

This underscores the biological fidelity of EscaPRRS as the model is not trained in explicit information on receptor or antibody binding. This correlation between interfacial maximum escape propensity sites and HADDOCK binding affinity trends with the seven receptors highlights potential antibody target sites. A comparison of binding affinity scores with the maximum site average scores with the interaction residues indicated a Pearson Correlation Coefficient (PCC) of 0.74 with a *P* value of 0.0569 (Fig. 6). We observed that high escape propensity residues recover 85.7% of the interfacial residues of GP5 with the seven porcine receptors considered. This result supports the hypothesis that immune evasion by steric effects, rather than improved receptor usage is the dominant selection pressure driving the evolution and emergence of pathogenic variants.

For such viral-host interactions, scaling binding affinity predictions are challenging as most tools often rely on structure predicting and geometry governed docking, wherein longer sequence length results in low confidence/disordered structures. To overcome this, we use our sequence-only binding affinity predictor tool, Seq2Bind (TwinPeaks module; ΔΔG tracker) (Dey and Chowdhury 2025, Ma *et al.* 2025) that maps protein language embeddings of the protein sequences to a latent space was used. We learnt and predicted GP5 variant affinity score (∼ΔΔG) trends and interfacial hotspot residues with all seven porcine receptors. A more negative binding affinity score (ΔG) is correlated with stronger binding between the viral protein and the porcine receptor. The ability to score binding affinities and antibody escape propensities at scale with Seq2Bind (Dey and Chowdhury 2025, Ma *et al.* 2025) offers a promising opportunity to incorporate EscaPRRS for concerted testing and surveillance of emerging/re-emerging strains and design constructs for immunogenicity.

It is important to note that antibody escape and receptor binding are independent events and are dictated by a range of host biology factors and non-modeled events like population-level effects, glycosylation, and host cell dynamics. This calls for integrative metabolic modeling in host systems incorporating viral entry, enzyme activity, metabolite transport, and molecular recognition, as outlined in our review (Noor *et al.* 2025).

### 3.4. Incorporating population and mutational dynamics for fitness prediction of variant sequences

**Figure 7:**
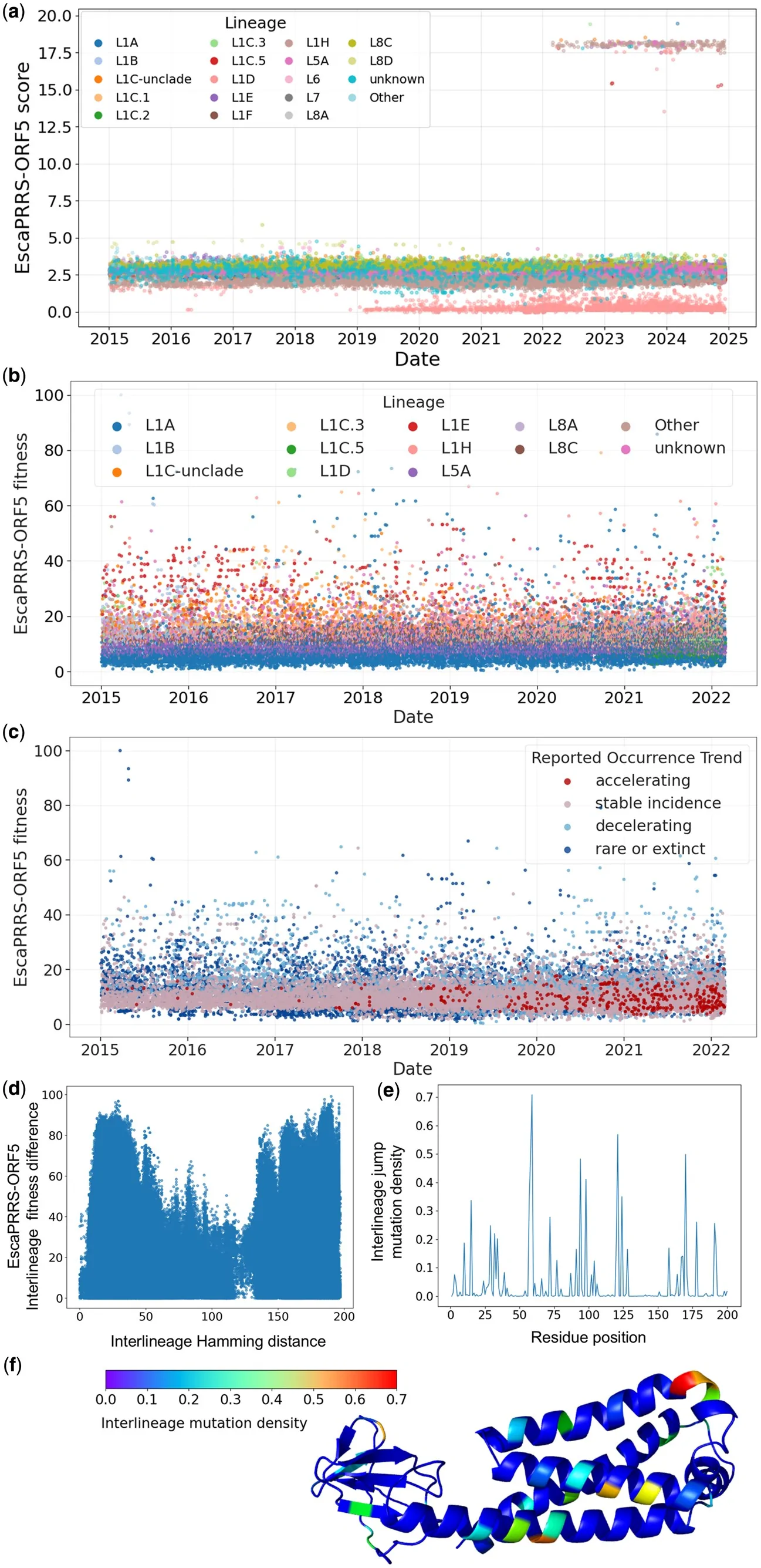
(a) Sigmoid sum of EscaPRRS-ORF5 scores of single point mutation in a sequence relative to reference calculated for all sequences in the local dataset over time, indicating the loss in capturing mutational variability beyond the sequence Hamming distance. (b) EscaPRRS-ORF5 fitness values calculated for each sequence from the ESM-2 embedding space learnt by the Bayesian VAE. (c) EscaPRRS-ORF5 fitness values colored based on population occurrence trends for the sub-lineages reported in PRRS-Loom webserver. Sub lineage wise temporal trends for the variants in the training dataset are provided in Supplementary file S3, available as supplementary data at *Bioinformatics* online. (d) Distribution of sequence edit distances versus fitness differences between two sequences classified into different lineages sampled across 4.18 billion inter-lineage pairs. (e) Density of mutation hotspots that drive lineage jumps for all sequences that have inter-lineage edit distances less than or equal to ten. (f) Lineage jump hotspots shown in (e) visualized on Alphafold-3 generated structure of PRRSV-GP5.

Given the complete mutational landscape, fitness prediction of complete sequences involves aggregating the effects of single point mutations. We infer that mere aggregation of the escape scores for the single point mutations that govern the fitness landscape to score a full sequence, as employed in EVEscape (Thadani *et al.* 2023), resulted in overdependency of the score on the number of mutations, which dilutes the effective predictive performance of the VAE in annotating fitness of any sequence from the latent space (Fig. 7, panel a). While traditional one hot encoding cannot represent a full sequence in a single embedding dimension, the use of protein language model embeddings allows a motif-informed representation of any sequence which can be conveyed and projected into the latent space for fitness predictions by ELBO difference relative to the reference sequence which captures the periodic and seasonal trends in variant fitness while agreeing with the sub-lineage occurrence trends reported in the PRRSLoom-Variants (n.d.) server (https://stemma.shinyapps.io/PRRSLoom-variants/) (Fig. 7, panel (b) and (c)). It is important to note that the lineage classification and RFLP typing are based solely on ORF5 and therefore do not fully explain the occurrence dynamics of variants in the population.

Since protein language model embeddings naturally capture higher order epistasis (Tang *et al.* 2026), our approach offers a significant leap over traditional methods that penalize fitness based on individual mutational effects to score full sequences for their epidemiologic fitness (Read *et al.* 2012) in highlighting the dynamic fitness landscapes in emerging/re-emerging variants within a sub-lineage.

ORF5-based variant classification (VanderWaal *et al.* 2025,  Zeller *et al.* 2024) of PRRSV-2 is quite powerful to pinpoint dominating lineages when inferred with sampling intensities to annotate variants of concern (VOCs). However, phylogenic analysis efforts do not detail on charting the mutations that shift a sequence from a lineage of lower reported pathogenicity to that of a VOC. To address this, we performed inter-lineage analysis (Fig. 7, panel (d)) by sampling 4.18 billion random pairs of sequences, each belonging to a different lineage, and comparing their sequence edit distances and fitness changes. This pinpoints mutation regions that drive lineage jumps by a small number of mutations between the two sequences. After filtering inter-lineage pairs where the sequences differ by less than or equal to 10 mutations, we identified hotspot regions that drive lineage jumps (Tewawong *et al.* 2017), as visualized in Fig. 7, panel (e) and (f). This included a Lysine at position 59, a Glutamine at position 58, and an Isoleucine at position 121 as the top three spots seen to be mutated to potentially shift to the next closest lineages (LIB, LIC) from L1A.

Thus, EscaPRRS-ORF5 gives a tractable static fitness score for each genotype, to highlight high fitness variants, which is inherently tied to a competitive replication in a population. When new sequences appear through mutation, fitness is counteracted by population growth, and the compensatory evolution requires a dynamic tracking. To address this, we apply a basic logistic population growth ODE model on EscaPRRS-ORF5 fitness for all re-emerging sequences in our dataset to follow multiple genotypes and their frequencies, as detailed in Supplementary File S2, available as supplementary data at *Bioinformatics* online. This formulation shall be enhanced with experimental data on growth and clearance rates for each lineage for reliable quantitative modeling of time-varying selection regimes (Read *et al*. 2012).

## 4 Conclusion

With rapidly evolving PRRSV infection patterns that challenge the current vaccine design paradigms, there is an urgent need to score and monitor emerging and re-emerging strains to enable timely interventions and strengthen preparedness for effective protection. While lineage classifications purely based on sequence similarity and phylogeny can help detect the spread and introduction of newer strains, fitness prediction is crucial to flag specific mutants and clades with higher propensity to immune escape and transmission. To our knowledge, there are no structure-aware tools available to predict and inform the epidemiological fitness for PRRSV mutants. We present EscaPRRS to score point mutations, complete variant sequences, and design constructs. A fundamental version of EscaPRRS is demonstrated in this work with PRRSV ORF5 sequence data, where we encode ESM-2 protein language model embeddings to a latent space to infer fitness of point mutants and sequences of the GP5 glycoprotein encoded by ORF5. ESM-2 is chosen for its open-source capabilities for downstream embedding processing, and its versatility in representing sequence motifs and non-additive mutational patterns without being trained on explicit structural features, which allows capturing subtle local mutational differences without being smoothened by the global fold information. Consequently, the current version of EscaPRRS-ORF5 is limited to GP5 sequences of length 200 (as usually observed in PRRSV-2), other protein language models including ESM-c can be leveraged to handle the effect of insertions and deletions.

ORF5 constitutes less than 5% of the whole PRRSV-2 genome and is widely characterized owing to rapid mutational variability and its role in antibody binding. To account for the structural and biochemical changes at the mutation sites that evade antibody binding, position-wise escape propensity scores are predicted for the complete mutational landscape (Supplementary Methods S1, available as supplementary data at *Bioinformatics* online), which can be extended to the complete genome to help identify critical antibody escape hotspots. This calls for a complete genome based PRRSV-2 classification, which when combined with EscaPRRS will provide scope for mapping potential hotspots for recombination, and domain-wise receptor binding epitopes.

The key takeaway of this work is not to argue that ORF5 alone provides a complete representation of PRRSV evolutionary dynamics, nor to suggest that an ORF5-centered model constitutes an endpoint of genomic surveillance. Rather, we establish a structurally informed, evolution-aware Esca toolchain that converts lineage-resolved sequence variation into mechanistic fitness and escape insight, forming a reusable and extensible framework that can integrate metagenomic coupling datasets (Hodges *et al.* 2025) and epitope–receptor interactomes to enable scalable, whole-genome, structure-aware surveillance of PRRSV-2 (or other viruses).

Overall, EscaPRRS-ORF5 allows surveillance of PRRSV-2 GP5 variants for fitness and identification of hotspots for receptor binding and antibody escape without being explicitly trained on any antibody or receptor information. EscaPRRS reduces the reliance on deep sequence alignments to assess the evolutionary patterns of emerging/re-emerging variants and escape vulnerability of design constructs that are antigenically similar to the emerging sequences, without heavy dependence on three-dimensional structures of antibody-antigen complexes. As a result, PRRSV-2 GP5 variants of concern can be flagged in advance as they emerge, to aid comprehensive experimental assays. Subsequent goals include incorporating EscaPRRS for an inverse design toolchain for constructing prototypic libraries of *de novo* antibodies or immunogens, as demonstrated in our other work (Teoh *et al.* 2025).

## Supplementary Material

btag529_Supplementary_Data

## Data Availability

While the training and test sequences used in this work are of proprietary nature, the PRRSView (Zeller *et al.* 2022) server shall be referred for a phylogenetic overview of PRRSV-2 *ORF5* GP5 sequences, including BLAST, RFLP scores, lineage and sub-lineage classification tools.

## Code availability

EscaPRRS is freely accessible at https://agrivax.studio suite of tools and within the StructF tab. Scripts to calculate EscaPRRS-ORF5 fitness scores are available at the following Google colab link: https://colab.research.google.com/drive/1TEgzAhPwvNAZ01VXeJbIFibfri2jnDA5? usp=sharing. Training and inference scripts for Esca are available in our Hugging face repository at https://huggingface.co/chowdhury-lab/escaprrs_demo for a generalized workflow which can be implemented for any viral protein sequence data. Additionally, EscaPRRS scores calculated as aggregated sigmoid sum escape scores of point mutations can be accessed at the following Google Colab link: https://colab.research.google.com/drive/1qs48k65aDkxuxO0rYcCDxtZPHjYlVMrO? usp=sharing.
